# Bone Regeneration Capability of 3D Printed Ceramic Scaffolds

**DOI:** 10.3390/ijms21144837

**Published:** 2020-07-08

**Authors:** Ju-Won Kim, Byoung-Eun Yang, Seok-Jin Hong, Hyo-Geun Choi, Sun-Ju Byeon, Ho-Kyung Lim, Sung-Min Chung, Jong-Ho Lee, Soo-Hwan Byun

**Affiliations:** 1 Department of Oral and Maxillofacial Surgery, Dentistry, Sacred Heart Hospital, Hallym University College of Medicine, Anyang 14068, Korea; kjw9199@hanmail.net (J.-W.K.); face@hallym.or.kr (B.-E.Y.); 2 Graduate School of Clinical Dentistry, Hallym University, Chuncheon 24252, Korea; 3 Department of Otorhinolaryngology-Head & Neck Surgery, Dongtan Sacred Heart Hospital, Hallym University College of Medicine, Dongtan 18450, Korea; enthsj@hanmail.net; 4 Department of Otorhinolaryngology-Head & Neck Surgery, Sacred Heart Hospital, Hallym University College of Medicine, Anyang 14068, Korea; pupen@naver.com; 5 Department of Pathology, Dongtan Sacred Heart Hospital, Hallym University College of Medicine, Dongtan 18450, Korea; byeon.sunju@welovedoctor.com; 6 Department of Oral and Maxillofacial Surgery, Dentistry, Korea University Guro Hospital, Seoul 08308, Korea; ungassi@naver.com; 7 R&D Center, Genoss, Suwon 16229, Korea; soodentist@naver.com; 8 Department of Oral & Maxillofacial Surgery, School of Dentistry, Seoul National University, Seoul 03080, Korea; leejongh@snu.ac.kr

**Keywords:** hydroxyapatite, tricalcium phosphate, 3D printing, digital light processing, customizable

## Abstract

In this study, we evaluated the bone regenerative capability of a customizable hydroxyapatite (HA) and tricalcium phosphate (TCP) scaffold using a digital light processing (DLP)-type 3D printing system. Twelve healthy adult male beagle dogs were the study subjects. A total of 48 defects were created, with two defects on each side of the mandible in all the dogs. The defect sites in the negative control group (sixteen defects) were left untreated (the NS group), whereas those in the positive control group (sixteen defects) were filled with a particle-type substitute (the PS group). The defect sites in the experimental groups (sixteen defects) were filled with a 3D printed substitute (the 3DS group). Six dogs each were exterminated after healing periods of 4 and 8 weeks. Radiological and histomorphometrical evaluations were then performed. None of the groups showed any specific problems. In radiological evaluation, there was a significant difference in the amount of new bone formation after 4 weeks (*p* < 0.05) between the PS and 3DS groups. For both of the evaluations, the difference in the total amount of bone after 8 weeks was statistically significant (*p* < 0.05). There was no statistically significant difference in new bone between the PS and 3DS groups in both evaluations after 8 weeks (*p* > 0.05). The proposed HA/TCP scaffold without polymers, obtained using the DLP-type 3D printing system, can be applied for bone regeneration. The 3D printing of a HA/TCP scaffold without polymers can be used for fabricating customized bone grafting substitutes.

## 1. Introduction

Guided bone regeneration (GBR) is used to increase the volume of the alveolar ridge or preserve the extraction socket. Currently, the bone graft materials used in GBR are classified as autogenous, allogenic, xenogeneic, and alloplastic bone [1,2]. Autogenous bone is regarded as the ideal material for restoring bone defects. It has advantages for new bone formation, such as osteogenicity, osteoconductivity, and osteoinductivity. However, some studies have reported disadvantages, such as donor site morbidity and limited availability [3,4]. Allografts and xenografts also have infection risks [5]. Among the alloplastic grafts, calcium phosphate bioceramic is mainly used because its composition is similar to that of bone mineral [6]. Biphasic calcium phosphate (BCP) ceramic is made up of hydroxyapatite (HA) and tricalcium phosphate (TCP) in different proportions [7]. HA can be fabricated into solid scaffolds with porosities for cell attachment, migration, and bone formation [8]. HA has attracted attention in the fields of bone tissue engineering, implant coatings, and the hybridization of materials [9]. The resorption rate of HA is slower than the rate of bone regeneration because of chemical stability [10]. To improve the resorption rate of HA, TCP, which has a fast resorption rate, has been introduced and suggested for use in combination with HA [10]. The composition and degradation of ceramic scaffolds are easily controlled, making them attractive scaffolds for use in bone regeneration [11].

Past studies showed how HA/TCP macropores were interconnected and induced growth into soft tissues and blood vessels. Fu et al. reported a ceramic scaffold with a proper compressive strength (166 MPa) comparable to that of cortical bone [12]. The scaffold had a large average pore size (1.2 mm) and high interconnectivity (100%) between the pores, with 60% porosity. Habibovic et al. showed a compressive strength value of 6.3 MPa for HA scaffolds, with 80% porosity and an average pore size of 450 μm; however, the interconnectivity between the pores was drastically compromised (~50%) [11,13]. Porosity allowed bone-forming proteins (BMP) to adhere on the surface of the material and induce bone formation [14,15,16].

This study compared the bone forming ability of 3D printed scaffolds versus particle-type bone, both of which are made up of this HA/TCP. Generally, particle-type or block bone substitutes are used for GBR. Block bone substitutes can provide better mechanical support than particle-type bone substitutes. Therefore, GBR sites with block bone substitutes show less collapse in the grafted area and better maintenance of their volume and contours than GBR sites with particle-type bone substitutes [17]. However, block bone substitutes usually need to be fixed and require additional manipulation, such as trimming to fit the defect area. When grafted block-bone substitutes are exposed, they are more likely than the particle-type bone substitutes to cause problems such as infection. To overcome the disadvantages of the two types of bone substitutes, demand for the manufacturing of customizable block bones has increased.

Personalized medicine is challenging for adapting the bone substitute to the patient’s specific bone geometry [18]. Customized block bones are made using computer-aided design and computer-aided manufacturing (CAD/CAM) or 3D printing [19]. The advantages of 3D printing include reduced material waste, optimizable surface and porous structures, and a reduced operation time [20]. For this reason, there is great demand for 3D printing technology, and many studies on 3D printing technology have been published [19,21,22]. 3D printing technology for block grafts is currently applicable to alloplastic materials. In general, BCP is widely used in 3D printing with synthetic polymers. Synthetic polymers are moldable to the required shapes and have excellent mechanical properties [23]. As the synthetic polymers degrade, they release acidic by-products that often lead to tissue necrosis and subsequent scaffold failure [24]. Therefore, it is desirable to remove these polymers before applying the grafts to the defect area. Using 3D printing technology, the block bone substitutes could be produced with the desired shape and structure. The 3D printed block bone substitutes would not need to be trimmed to fit the defects, which could shorten the operation time and result in good suitability for the defect area.

In this study, the HA/TCP scaffold was manufactured without polymers using 3D printing. To our knowledge, animal experiments for evaluating the bone regenerative ability of 3D printed HA/TCP scaffolds without polymer are lacking. Our hypothesis is that 3D printed HA/TCP scaffolds without polymer will have the required mechanical strength to endure the pressure from the surrounding structure and sufficient biocompatibility for enhancing bone regeneration. This study was conducted to evaluate the bone regeneration of the customizable HA/TCP scaffold without polymers using digital light processing (DLP)-type 3D printing.

## 2. Materials and Methods

Twelve healthy adult male beagle dogs that were approximately 22–26 weeks old and 10–12 kg in weight were enrolled for this study. The study protocol was approved by the Animal Ethical Committee for Animal Research at Genoss^®^ (Suwon, Korea), (GEN-IACUC-1908-01). Before the experiment, the dogs had free access to food and water and were given one week to become accustomed to their new environment prior to the operation.

### 2.1. Manufacture of 3D Printed HA/TCP Scaffold

**Figure 1:**
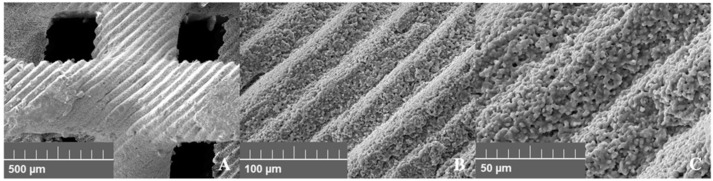
Structure of 3D printed hydroxyapatite (HA)/tricalcium phosphate (TCP) scaffold (SEM, 20.0kV). (**A**) 100×, (**B**) 500×, (**C**) 1000×.

**Figure 2:**
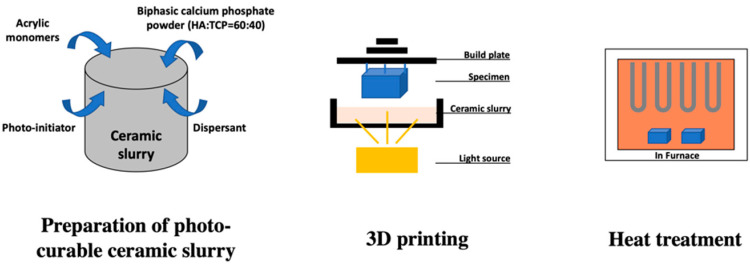
Digital light processing (DLP)-type 3D-printing process for pure HA/TCP scaffold.

The 3D printed scaffold substitute was a synthetic bone graft material produced by a DLP-type 3D printer (Cubicon Lux, Cubicon^®^, Sungnam, Korea). The 3D printer had a resolution of 100 µm and could print with a thickness of 20–100 µm (Figure 1). After designing the bone graft material for the defect size (7 × 3 × 5 mm), the design data were converted into a stereolithography (STL) file. The file was printed with the 3D printer using a photoreactive ceramic-resin composite. The slurry was made up of HA/TCP (6:4 ratio), a dispersant, acrylic monomers, and a photo initiator. The resin component was removed completely through heat treatment after 3D printing (Figure 2). The detailed method of the manufacture of 3D printed HA/TCP scaffolds without polymer is a trade secret of Genoss^®^, so the procedure and technique cannot be described in this article.

### 2.2. Compression Test of 3D Printed HA/TCP Scaffold Block

The compressive strength of the specimens was evaluated (ASTM D695). After specimen placement, the rod of the universal testing machine (H50K-T UTM^®^, Tinius Olsen corp., Surrey, UK) was lowered at a rate of 0.5 ± 0.1 mm/min, and the load applied to the sample was measured. The extracted load data were calculated per the distance divided by the cross-sectional area of the specimen. The values were plotted as distance (µm, linear scale) versus stress (N/cm^2^, log scale). The compression stress values were derived from the intersection of rapid and gradual stress increases.

### 2.3. Cytotoxic Test of 3D Printed HA/TCP Scaffold Block

The in vitro cytotoxicity test standard (ISO 10993-12) was used to determine the cytotoxicity of the specimen (three specimens each). The 4g 3D printed HA/TCP specimen was eluted for 24 h at 37 °C in 20 mL of elution solvent. Meanwhile, 500 mL of minimal essential medium was prepared by mixing 50 mL of fetal bovine serum (Gibco^®^, Thermo Fisher Scientific, Green Island, NY, USA) and 10 mL of penicillin–streptomycin solution (Welgene^®^, Gyeongsan-si, Korea). High density polyethylene and natural rubber were used as negative and positive control materials, respectively. The control solutions were eluted under the same conditions. Mouse fibroblasts (ATCC CCL 1, clone 929 of strain L, Korean Research Institute of Bioscience and Biotechnology, Daejeon, Korea) were injected into a flask containing the antibiotics and fetal bovine serum, and the flask was maintained in an incubator at 37 °C. The culture solution in the flask was changed three times a week.

One day after injecting the cell culture medium into a 6-well plate, the cells had attained 80% confluence. After the complete removal of the cell culture medium, 2 mL each of the elution solvent of the negative control, positive control, and experimental specimen was applied to and cultured in the 6-well plate. After culturing for 24 h and 48 h, cell growth and lysis levels were measured using a microscope. The condition of the cells was evaluated by the grade of the cells. When the grade of the negative control eluate was zero and that of the positive control eluate was higher than 4, the test was considered reliable. If the grade of the experimental specimen was lower than 2, no cytotoxicity occurred (Table 1).

## 3. Surgical Procedure

General anesthesia was performed intravenously with 0.1–0.14 mL/kg of xylazine and 0.01 mg/kg of tiletamine–zolazepam. Orotracheal intubation was performed using size 6 tubes without ballooning for securing the airway. The tube was fixed to the anterior part of the mandible using paper tape, and the fixed part was marked so that the position of the tube remained constant. Upon intubation, anesthesia was maintained using isoflurane (Piramal Critical Care, Inc., Bethlehem, PA, USA) and oxygen by inhalation. After the operation, each animal received analgesic medication (meloxicam, 0.2 mg/kg) and antibiotic medication (enrofloxacin, 0.2 mL/kg) through intramuscular injection for seven days. Each animal was fed a liquid diet for one week after the operation.

### 3.1. First Surgery for Tooth Extraction

After the crestal incision, the mucoperiosteal flaps were elevated bilaterally from the mandibular first premolar (P1) to the first molar (M1), and the teeth from P1 to M1 were carefully extracted on both sides. X-rays were taken to identify the existence of retained roots in the extraction sockets. Finally, the flap was repositioned and sutured with nylon.

### 3.2. Second Surgery for Preparation of Defect (GBR)

After 10 weeks, incision and flap elevation were done similarly to prepare the defects for GBR. The bone defects were made using a prepared defect guide (7 × 3 × 5 mm^3^) and high-speed dental burs. A total of 48 defects were created, with two defects on each side of the mandible in all the dogs. The defect sites for the positive control groups (PS group, sixteen defects) were filled with a particle-type substitute (OSTEON 3, Genoss^®^, Suwon, Korea), whereas defect sites for the experimental groups (3D printed substitute (3DS) group, sixteen defects) were filled with a 3D printed substitute grafted bone. The defect sites for the negative control group (NS group, sixteen defects) were left untreated for spontaneous healing. The grafted bone and defect sites were covered with a collagen membrane (Collagen Membrane, Genoss^®^, Suwon, Korea). Fixation pins (Membrane Pin, Dentium^®^, Suwon, Korea) were used for stabilizing the grafted bones and membranes. Finally, the flap was repositioned and sutured using 4-0 Vicryl (Vicryl^®^, Ethicon, Somerville, NJ, USA). The sutures were removed two weeks after the second surgery.

The dogs were sacrificed after healing periods of four and eight weeks. Six dogs were sacrificed after each healing period. The mandibles were dissected, and the experimental specimens were harvested. All the specimens obtained were fixed in a 10% neutral buffered formalin solution.

## 4. Radiological Examination

X-ray microcomputed tomography was conducted using a Skyscan 1173 (Bruker-micro CT, Belgium) four and eight weeks after sacrifice. After processing in a CT Analyzer 1.13 (Bruker-micro CT, Belgium), the X-ray transmittance intensity of the remaining grafted materials and new bone was analyzed. The percent bone volume was calculated using the following formula:

Percent bone volume (%) = bone volume/tissue volume × 100.

## 5. Histomorphometric Examination

The fixation of the specimens was performed in neutral buffered formalin at a temperature of 4 °C for one week. A tissue slide was made by making a longitudinal section through the bone tissue. A total of 48 slides were stained with H&E/Goldner’s trichrome staining.

The slide scanning was performed with a digital slide scanner (Pannoramic 250 Flash III, 3DHISTECH Ltd. Budapest, Hungary). The digital images were evaluated and quantified using histomorphometrical measurements. The examination was performed with a 200× magnified microscope and CaseViewer (3DHISTECH Ltd., Budapest, Hungary). The digital image was evaluated using Image-Pro Plus (Jenoptik, Seoul, Korea), and the data were summarized in Excel. The percentage of new bone was calculated using the following formula:

New bone (%) = [Area of new bone/Total area of defect] × 100.

## 6. Statistical Analysis

The statistical analysis was conducted using the Statistical Package for the Social Sciences (Version 12.0K; SPSS, Inc., Chicago, IL, USA). The differences between three groups were evaluated with the Kruskal–Wallis-test. Comparisons between groups were performed with the Mann–Whitney U test, and results with *p*-values < 0.05 were considered as statistically significant.

## 7. Results

### 7.1. Compression Test of 3D-Printed HA/TCP Scaffold Block

A total of 16 specimens were evaluated. The compressive strength of the HA/TCP scaffold block was 2.80 ± 0.8246 MPa.

### 7.2. Cytotoxic Test of 3D Printed HA/TCP Scaffold Block

At 24 h and 48 h, the grades of cells using the negative and positive control eluate were zero and four, respectively. The eluate of the control showed no change. The grades of the cells in the HA/TCP specimens were zero at both 24 and 48 h, thereby confirming no cytotoxicity (Table 2).

### 7.3. Clinical Evaluation

No dog in any group was lost during the experimental period. None of the groups showed specific problems such as inflammation, wound dehiscence, swelling, or infection. The collagen membrane overlying the bone graft material was not absorbed completely at 4 or 8 weeks.

### 7.4. Radiological Evaluation

**Figure 3:**
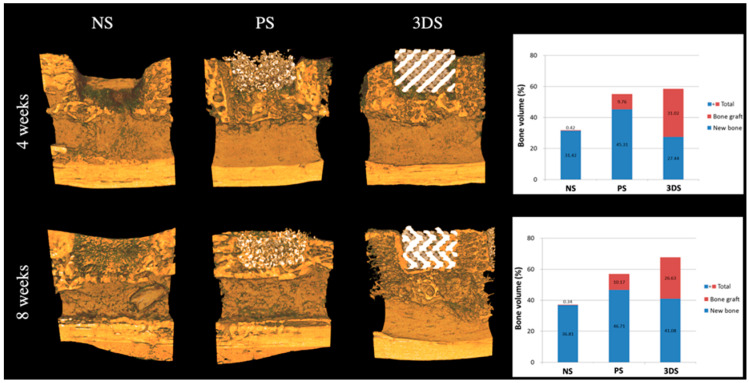
Radiological evaluation at 4 and 8 weeks.

At 4 weeks, the percentage formation of new bone was 45.31 ± 3.74% in the PS group, and 27.44 ± 5.86% in the 3DS group. The remaining bone in the grafted material was 31.02 ± 1.35% in the 3DS group (Figure 3). Table 1 shows that the new bone formation in the 3DS group was 41.08 ± 3.96% at 8 weeks. At 4 and 8 weeks, the total amounts of remaining and new bone in the 3DS group after GBR were 58.47 ± 6.36% and 67.72 ± 11.25%, respectively (Figure 3).

The NS and PS groups had a significant difference in the amount of new bone formation at 4 weeks. A statistically significant difference between the PS and 3DS groups at 4 weeks (*p* < 0.05) was observed (Figure 3). The PS group showed a larger amount of new bone formation at 4 weeks than that of the NS and 3DS groups. Moreover, the total amount of bone showed a statistically significant difference between the NS and PS groups and between the NS and 3DS groups at 4 weeks (*p* < 0.05) (Figure 3). The 3DS and PS groups showed higher values of the total amount of bone than the NS group at 4 weeks. However, no statistically significant difference was shown between the PS and 3DS groups in the total amounts of bone at 4 weeks (*p* > 0.05).

There were no significant differences in the amounts of new bone at 8 weeks among all the groups. At this time, the total amount of bone showed a statistically significant difference between the NS and PS groups and between the NS and 3DS groups (*p* < 0.05) (Figure 3). A statistically significant difference in the total amount of bone was observed between the PS and 3DS groups at 8 weeks as well (*p* < 0.05). The three groups from the greatest to least relative total amounts of bone were 3DS, PS, and NS.

### 7.5. Histomorphometric Evaluation

There were no specific inflammatory reactions associated with foreign body reactions, including necrosis and granuloma, in any of the groups. There were no other specific reactions in any group.

**Figure 4:**
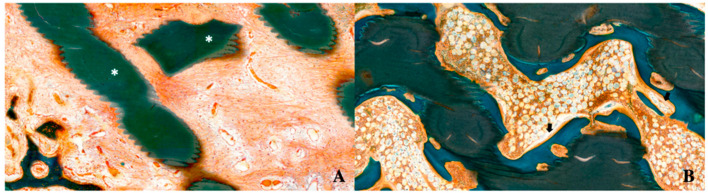
Histomorphometric evaluation at 4 and 8 weeks in the 3D printed substitute (3DS) group. (**A**) After 4 weeks. (**B**) After 8 weeks (white asterisk: grafted bone; black arrow: osteoblast).

In the PS and 3DS groups, granulation tissue was observed in the defect area at 4 weeks, but little or no granulation tissue was observed at 8 weeks; however, hematopoietic cells were observed. Vascularization was observed at 4 and 8 weeks in the PS and 3DS groups. New bone formation was hardly observed at 4 weeks; however, it was easily observed at 8 weeks (Figure 4).

**Figure 5:**
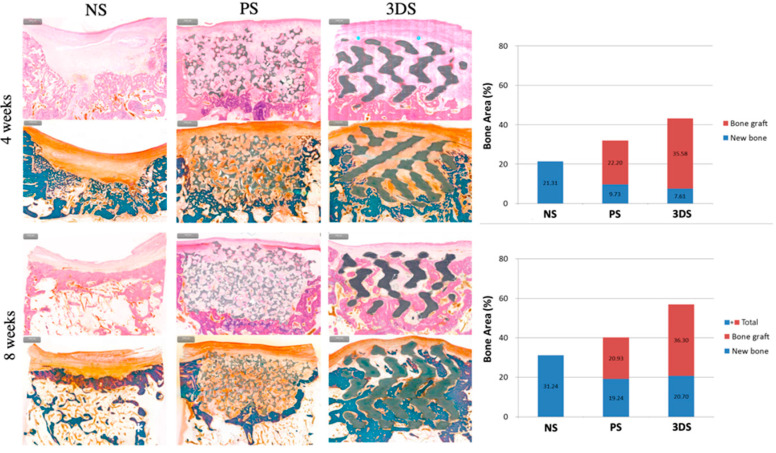
Histomorphometric evaluation at 4 and 8 weeks.

The 3DS group clearly showed the ingrowth of new bone from the lower side to the center at 8 weeks. In the PS group and 3DS groups, more ingrowth of new bone was observed at 8 weeks than at 4 weeks (Figure 5). Figure 5 shows that at 4 weeks, the amount of new bone formation was 9.73% and 7.61% in the PS and 3DS groups, respectively. In addition, at 8 weeks, the amount of new bone formation was 19.24% and 20.70% in the PS and 3DS groups, respectively. At 4 weeks, the new bone formation of the PS group showed a statistically significant difference compared to that of the NS group, and the 3DS group showed a statistically significant difference compared to the NS group (*p* < 0.05). The PS group and 3DS group showed lower values of new bone than that in the NS group at 4 weeks. There was no statistically significant difference in new bone between the PS group and the 3DS group (*p* > 0.05). Statistically significant differences were shown among the groups regarding the total amount of bone (*p* < 0.05). The 3DS group showed a higher value of the total amount of bone than the NS group and PS group, and the PS group showed a higher value of total amount of bone than the NS group at 4 weeks.

At 8 weeks, the new bone formation of the PS group and 3DS group was statistically different from that of the NS group (*p* < 0.05). The new bone formation of the PS group and 3DS group was lower than that of the NS group. There was no statistically significant difference in new bone between the PS and 3DS groups (*p* > 0.05). Statistically significant differences in the total amount of bone (*p* < 0.05) were observed between the NS and PS groups and between the PS and 3DS groups. At 8 weeks, the total amount of bone was the highest in the 3DS group, followed by that in the PS group and then that in the NS group.

## 8. Discussion

In non-segmental defects, there were five different types of defect: rectangular, box, arc, saddle, and cylindrical [25]. Calculating the critical size defect in order to compare different sizes is difficult because all the experimental animals were exterminated at different points in time [25]. Previous studies set the defect size as 3 × 5 mm for the rectangular defects and 8 × 12 mm for the box-type defects in a mandible model of a beagle dog [25,26]. If the defect size is large, the possibility of complications—such as mandible fracture, bleeding, and infection—increases. Based on previous studies, this study set the defect size as 7 × 3 × 5 mm^3^, but there was new bone formation at 4 weeks and 8 weeks in the negative control group. Huh et al. reported that, when the periosteum was preserved, the defect length in the beagle dog needed to be greater than 50 mm in order to fail to heal [27]. During the surgical procedure of this study, the periosteum was preserved; thus, new bone formation could occur.

It was assumed that the bone formation percentage for the 3D printed HA/TCP scaffold would be lower than that for the particle-type bone substitutes; however, this was not the case. This study calculated the new bone formation as follows: New bone (%) = [Area of new bone/Total area of defect] × 100. The total area of the defect was the same in the three groups (7 × 3 × 5 mm^3^). However, the new bone could only be formed in the remaining area of the grafted material area in the defect, since the grafted ceramic scaffold would not be absorbed within 8 weeks. Thus, the PS and 3DS groups had smaller areas for generating the new bone than the NS group. Although the rest area in the PS and 3DS groups was smaller than that in the NS group, the new bone formation of the PS and 3D groups was greater than that of the NS group as observed in radiological evaluation at 8 weeks. The amount of remaining grafted material at 8 weeks was found to be the highest in the 3DS group. Moreover, the 3DS group maintained the largest volume of grafted material of all the groups at 4 and 8 weeks. The PS and 3DS groups showed vascularization after 4 weeks. Kuboki et al. observed higher bone formation in porous hydroxyapatite scaffolds, with pore sizes in the range of 300–400 μm after 4 weeks of implantation in rats [28]. This result was explained by the rapid vascularization within the implanted scaffolds, which provided a proper microenvironment for osteogenesis. These results indicate that the average pore size of scaffolds can be manipulated to potentially improve the formation of bone and vascular networks in ceramic scaffolds [28].

Guided bone regeneration is conducted with bone substitutes to prevent volume loss or to increase the bone width and height [29,30,31]. There are many studies comparing block-type autogenous bone and particle-type allogenic bone, and most studies have demonstrated that the absorption rate of block-type bone substitutes is low [32,33,34,35]. It is known that the use of block bone substitutes is more effective for large defects than particulate substitutes because the block bone can properly support the surrounding structure [36]. However, when the block bone substitutes cannot fit the defect site well [6,37,38], 3D printing technology has made it possible to preoperatively create customized shapes as desired [39,40]. Using this technology, the block-type bone can be custom-manufactured with the appropriate size and shape. Polymers are mainly used as the material in 3D printed bone substitutes. However, the molecular weight of the polymer—which causes incompleteness in pore size, porosity, and interconnectivity—negatively affects cell adhesion to the scaffold [41,42]. In general, it is known that polymers have less bone conduction ability than calcium phosphate-based artificial bone [43]. HA/TCP is a suitable ceramic material for manufacturing bone-grafting materials using 3D printing. It is known to exhibit excellent biocompatibility, biodegradation, osteoconductivity, and osteoinductivity [44,45]. In addition, the osteoinduction property depends on the surface chemistry and charge of calcium phosphate ceramics, which can influence protein adsorption and, in turn, promote cell differentiation through cell–extracellular matrix interactions [46]. The resolution, porosity, and strength of 3D printed TCP scaffolds strongly depend on the particle size, depowering efficiency, binder droplet size, and scaffold geometry [47]. Beta-TCP is the most favorable form of TCP scaffold, owing to its mechanical strength and chemical stability.

Most scaffolds with HA/TCP are fabricated by conventional methods such as gas foaming, fiber bonding, freeze drying, phase separation/inversion, and particulate leaching [48]. However, the pore shape, geometry, porosity, and interconnectivity of the scaffolds cannot be controlled by these methods, and the fabricated scaffolds cannot be specifically adapted to promote cell growth and tissue regeneration [49]. To overcome the limitations of conventional methods for the fabrication of ceramic scaffolds, 3D printing technology has been widely used [50]. There are various types of 3D printing systems in the biomedical field. Most previous studies used fuse deposition modeling (FDM)-type 3D printing to manufacture bone material [51,52]. FDM is usually performed by mixing HA or TCP powders with polymers and stacking them through a nozzle. It is necessary to use polymers—such as polycaprolactone, polylactic acid, and poly-DL-lactic acid—that melt at high temperatures as a solvent for TCP to maintain the shape and structure of the 3D printed ceramic materials. HA/TCP material with polymer is inferior in terms of its biocompatibility and new bone-forming ability due to the polymer [53,54]. In addition, the nozzle diameter of an FDM-type 3D printer is approximately 0.4 to 0.5 mm; therefore, it cannot print the bone material precisely and in detail.

Very few studies have reported the fabrication of HA/TCP scaffolds using a DLP-type 3D printing system. Unlike FDM-based 3D printers, the DLP-type 3D printing system used herein reduces the processing time by conducting the simultaneous irradiation of the entire desired cross-section. It can produce biomaterial with a high resolution compared to other systems [55]. DLP technology uses a digital projector as a light source, which significantly reduces the printing time and enables high-accuracy printing [56]. A DLP system uses a ceramic slurry stacked layer by layer for the production of ceramic scaffolds [57,58]. Light-unresponsive HA and TCP should be mixed with other polymers for DLP printing [59] because each layer is solidified by exposure to light. Subsequent heat treatment is used to remove the polymers completely and produce biocompatible substitutes. In DLP-type 3D printing systems, developing a technology that can completely remove the photopolymer by heat treatment after printing is key [60]. There are several essential conditions that need to be satisfied for complete removal, such as an appropriate heating rate, temperature, and time. The methods for removing polymers, methods for making the HA/TCP slurry, and 3D printing sequences are trade secrets in the field of bioceramic 3D printing. In this study, the safety and appropriateness of 3D printed bone were verified through biological safety assessments.

This study showed the possibility to manufacture 3D printed HA/TCP bone with complete polymer removal after 3D printing. This study also proved the biocompatibility and bone regenerative abilities of the 3D printed bone substitutes with the same composition as that of conventional HA/TCP alloplastic bone but with additional structural stability.

The bone regeneration and biocompatibility of the 3D printed HA/TCP scaffold were confirmed through this study. Further studies on scaffold structure and porosity in 3D printed bone are needed.

## 9. Conclusions

In this study, a 3D printed HA/TCP scaffold was fabricated using a DLP-type 3D printer. The 3D printing of HA/TCP scaffolds using a DLP system is rare and innovative, and it will enable customized bone grafting for biomedical applications. However, this study has a few limitations. The mechanical strength and bone regenerative ability of the 3D printed ceramic scaffold could be affected by the pore structure. Further study is required for accurately determining the pore size, scaffold geometry, and interconnectivity. In addition, growth factor and bioprinting techniques could enhance the bone regeneration facilitated by the ceramic scaffold. This study did not include those techniques; therefore, additional research will be helpful for increasing the utility of 3D printed HA/TCP scaffolds without polymer.

A 3D printed HA/TCP scaffold without polymers had sufficient bone-forming ability and provided superior stability for the defect area. The 3D printed HA/TCP scaffold is easy to use for large or complex bone defects. The 3D printing of a HA/TCP scaffold without polymers could be used for fabricating customized bone-grafting substitutes.

## Figures and Tables

**Table 1:** Reactivity grades for elution test.

Grade	Reactivity	Condition of All Cultures
0	None	Discrete intracytoplasmic granule, cell lysis.
1	Slight	No more than 20% of the cells are round, loosely attached, and without intracytoplasmic granules; occasional lysed cells are present.
2	Mild	No more than 50% of the cells are round and devoid of intracytoplasmic granules; extensive cell lysis and empty areas between cells.
3	Moderate	No more than 70% of the cell layers contain rounded cells and/or are lysed.
4	Severe	Nearly completely destruction of the cell layers.

**Table 2:** Results of cytotoxicity tests.

	Cytotoxicity Grades								
	Initial			24 h			48 h		
Experimental	0	0	0	0	0	0	0	0	0
Negative control	0	0	0	0	0	0	0	0	0
Positive control	0	0	0	4	4	4	4	4	4
